# On the Non-Thermal Mechanisms in Microwave Sintering of Materials

**DOI:** 10.3390/ma18030668

**Published:** 2025-02-03

**Authors:** Ming-Syun Lin, Kwo-Ray Chu

**Affiliations:** Department of Physics, National Taiwan University, Taipei 106, Taiwan

**Keywords:** microwave sintering, low-temperature densification, ponderomotive forces, magnetism-created cohesive forces, polarization-enhanced neck growth, polarization charge-enhanced mutual attraction

## Abstract

The microwave sintering of various materials is a promising technology, which has received much attention for its demonstrated potential. Both the conventional (furnace) and microwave sintering rely on thermal activation for particle bonding, for which a high temperature environment is essential. In comparison, microwave treatment achieves the same degree of densification as furnace sintering in a time shorter by a factor of two or higher and at a temperature lower by 5% to 15%. However, this is a phenomenon not yet fully understood and is commonly referred to as a non-thermal effect. Its understanding is a subject of both physics and practical interest. The non-thermal effect has been studied under years of research in order to broaden the applicability of microwave sintering. Here, we first present an overview of experimentally demonstrated advantages of microwave sintering. To facilitate further studies, we then review the literature and put together four commonly recognized interpretations of the non-thermal effects: the ponderomotive force-driven mass transport, magnetism-created cohesive forces, polarization charge-enhanced wave electric field, and polarization charge-induced attractive force among the sintered particles, with an emphasis on recent development.

## 1. Introduction

Sintering has been a widely used technique to densify fine powders into desired materials. In the process, thermal energy causes the particles to bond and the necks between particles to grow into a densified material [1]. The furnace remains the dominant source of heat. However, microwave heating started upon the availability of high-power microwave applicators in the 1960s and has produced encouraging results. A recent review by Ćurković et al. [2] presented the basic theory and progress made in the microwave sintering of metals, alloys, ceramics, composites, and glasses. Other reviews put emphasis on the fundamentals, advantages, applications [3], equipment development and commercialization [4], the range of materials sintered by microwaves [5], modeling [6], susceptor-assisted sintering [7], microwave-associated heating strategies [8], and the sintering of metal matrix composites [9]. A more recent review by Batienkov et al. [10] focused on recent studies in sintering metal powders and composite materials

**Figure 1:**
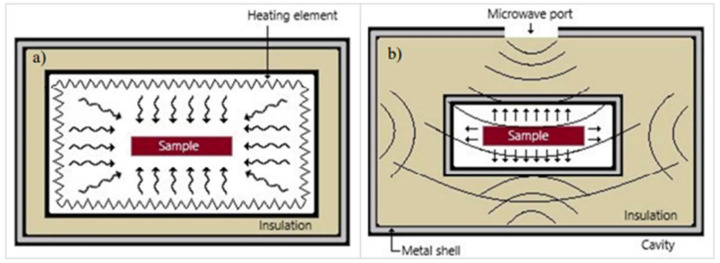
Illustration of heat flow in (**a**) a furnace and (**b**) a microwave oven [11]. Reproduced with permission from Elsevier, 2025.

As illustrated in Ref. [11], in contrast to the surface absorption and slow heat conduction into the center of the material in a furnace (Figure 1a), microwave heating is volumetric in nature (Figure 1b), resulting in much faster heat deposition. Equally important, the applicator walls (typically a conductor) are heated by microwaves at a rate ~50 times smaller than by the infrared radiation in a furnace [12], hence allowing significant energy saving due to much lower black-body radiation leakage out of the applicator.

Microwave sintering applies to a variety of materials. Ref. [2] lists the type of materials that have undergone significant improvements under microwave sintering. The sintered materials can be classified into four categories: 1. ceramics (mainly inorganic materials), 2. composite materials (of metal, ceramic, polymer, or multi-phase materials), 3. metal or alloy (two or more chemical substances mixed to form a substance with metallic properties), and 4. others (such as glass).

Microwave sintering is also a technology with wide application potential. Reference [3] enumerates the following five advantages of microwave sintering:

(1) Lower energy consumption.
(2) Higher heating rates or shorter heating time.
(3) Intensified diffusion process.
(4) Better grain distribution at higher density.
(5) Improved mechanical and physical properties.

Microwaves lie between 300 MHz and 300 GHz. The 2.45 GHz microwave is used in the vast majority of experiments, while millimeter waves can produce even more rapid heating [13,14]. There are, however, also problems reported, such as a sudden rise in the temperature of ceramic materials (thermal runaway) [15,16], stages in the process of thermal runaway [17], the region of thermal stability [18], and the method of reliable performance [19]. Sample fractures are another reported problem [20]. To date, microwave sintering still needs better characterization and control for broader industrial acceptance.

One of the less-understood issues in microwave sintering happens to be its biggest advantage. It is well documented that microwave sintering leads to a higher density at the same temperature [2,3,21,22,23,24,25,26]. For example, Brosnan et al. sintered alumina by the two methods for a comparison. At 1500 °C, microwave and furnace heating achieved a density of ~100% and ~70%, respectively [21]. From another angle, when reaching the same density, microwave sintering requires a lower temperature, as has been observed in numerous experiments [27,28,29,30,31]. In some cases, the microwave is the exclusive source for material treatment, such as the microwave spin resonance investigation [32].

This article will focus on the higher densification phenomenon in microwave sintering and review the proposed mechanisms for it. There are currently several theories as to why this occurs: 1. ponderomotive force-driven mass transport [33,34,35,36,37], 2. magnetism-created cohesive forces [38,39,40], 3. polarization charge-enhanced wave E-field [41,42], and 4. polarization charge-induced attractive forces among the sintered particles [43,44]. These are generally referred to as non-thermal effects (or microwave effects). However, at present, the reasons for such non-thermal effects are still inconclusive.

To probe further, an overview of a large volume of the documented literature appears warranted. In Section 2, we begin with a review of observations of the non-thermal effects, followed in Section 3 with a survey of early theories on the causes of these effects. Then, in Section 4, we put our emphasis on the polarization charge effects. The conclusion is presented in Section 5.

## 2. Observations of the Non-Thermal Effects in Microwave Sintering

Low-temperature densification occurs in the microwave sintering of various materials. Some examples are discussed below.

### 2.1. Ceramic Material

**Figure 2:**
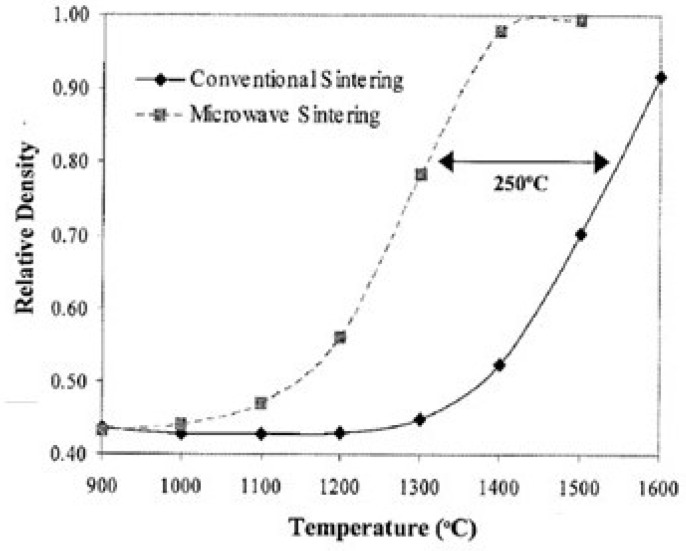
Alumina powder density comparison between furnace and microwave sintering at different temperatures [21]. Reproduced with permission from Elsevier, 2025.

Brosnan et al. [21] compared the densification of alumina powder sintered in the furnace and microwave at different temperatures. The sample consists of high-purity alumina (35 wt% transitional γ-alumina and 65 wt% α-alumina, doped with 500 ppm of MgO and 350 ppm of Y_2_O_3_). Figure 2 shows that, when reaching the density of about 0.8, the temperature required for microwave sintering is lower by ~250 degrees.

### 2.2. Metal Powder

**Figure 3:**
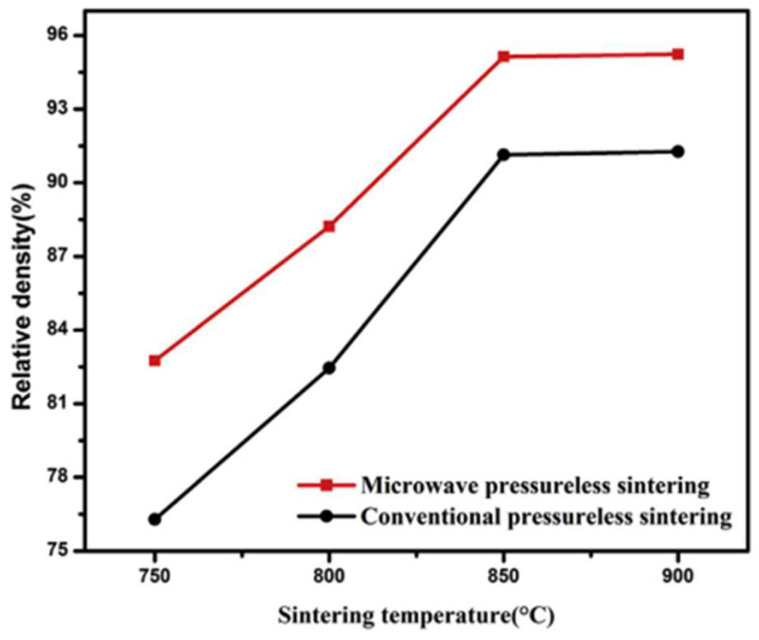
Densities of FeCuCO metal powder under furnace and microwave sintering versus the temperature [22]. Reproduced with permission from Elsevier, 2025.

Wang et al. [22] compared the densification of FeCuCO metal powder (see Table 1 for composition) between furnace and microwave sintering. Figure 3 shows that microwave sintering achieved a higher density at the same temperature. In other words, to achieve the same density, the required temperature is lower in microwave sintering. Furthermore, the ultimately achieved density is higher by microwave sintering.

### 2.3. High Magnetic Permeability Ferrite

**Figure 4:**
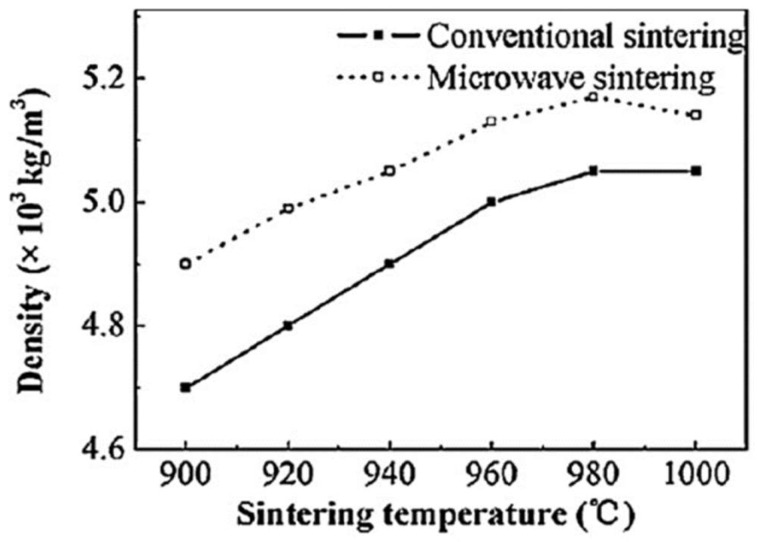
Temperature dependence of high-permeability ferrite density under furnace and microwave sintering [23]. Reproduced with permission from Elsevier, 2025.

In a similar example, Yan and Hu [23] compared the results of the furnace and microwave sintering of high-permeability ferrite. Figure 4 shows that, at the same density, the temperature required for microwave sintering is lower. Again, the ultimately achieved density is higher by microwave sintering.

### 2.4. Composites

**Figure 5:**
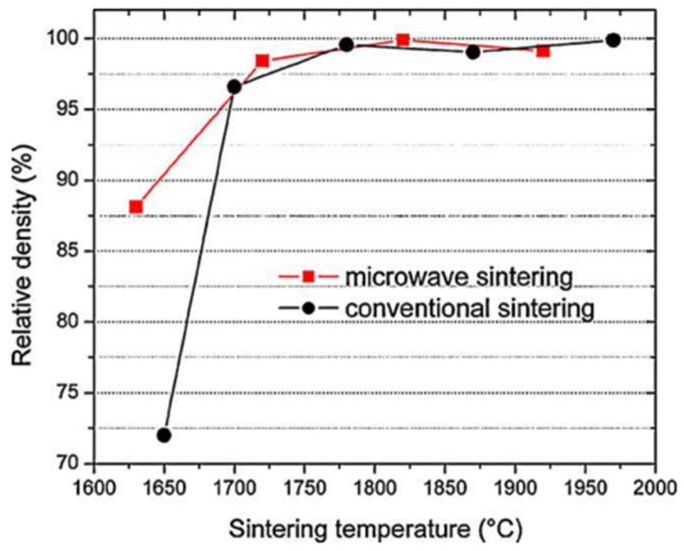
Density comparison of ceramic composites (ZrB_2_–4 wt.% B_4_C) sintered in a furnace and microwave oven versus the temperature [24]. Reproduced with permission from Elsevier, 2025.

Another comparison was reported by Zhu et al. [24] on the densification of a ZrB_2_–B_4_C particulate ceramic composite under furnace and microwave sintering (Figure 5). In this experiment, ZrB_2_ absorbs little microwave energy. Hence, 4 wt.% of B_4_C powder (a high-absorption material) was added to facilitate microwave absorption. The results show that both processes lead to almost full densification at 1700 °C or higher, but microwave sintering clearly exhibits a non-thermal effect with more effective densification at lower temperatures.

### 2.5. Crystallization

**Figure 6:**
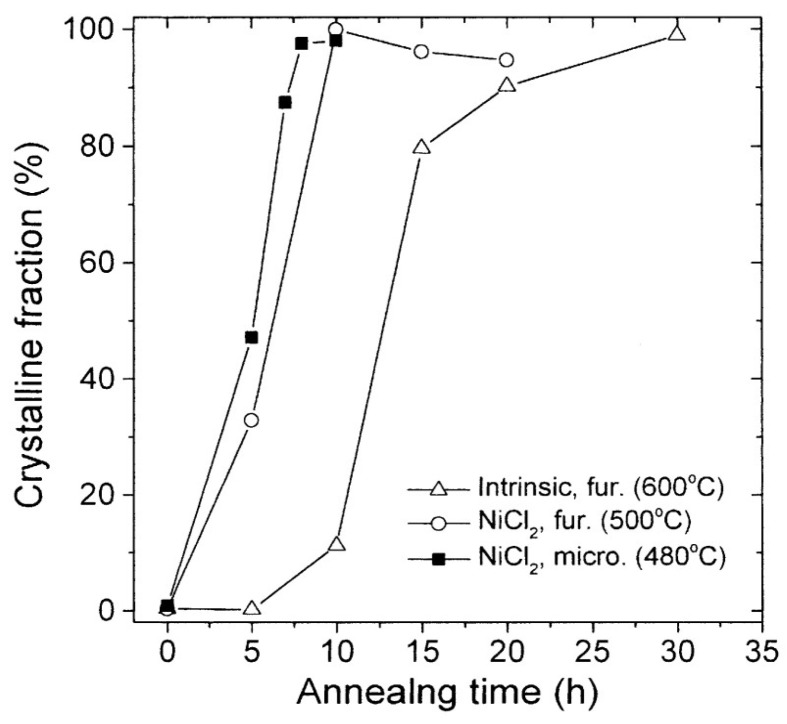
Comparison of the crystallization of an intrinsic amorphous NiCl_2_ sample, with and without a-Si film coating, annealed in a furnace and microwave oven [31]. Reproduced with permission from Elsevier, 2025.

Ahn et al. [31] found that microwave annealing of the metal coating of amorphous Si (a-Si) led to crystallization at a lower temperature than in furnace heating, while microwave heating further enhanced the crystallization. As shown in Figure 6, fully crystallization of the a-Si film at a 600 °C furnace requires about 30 h. However, a NiCl_2_-coated a-Si film was fully crystallized much sooner (10 h) and at a lower temperature (500 °C). In microwave heating, the NiCl_2_-coated a-Si film was fully crystallized in an even shorter time (8 h) at a still lower temperature (480 °C).

## 3. Early Theories on Causes for the Non-Thermal Effects in Microwave Sintering

There are several proposed reasons as to why microwave sintering causes low-temperature densification. Some are due to the wave E-field; some due to magnetic effects. The diversity of theories is due to the variety of materials processed in different ways. Thus, there may not be a single theory applicable to all cases. For these reasons, below, we sample the main points of these theories, beginning with the ponderomotive diffusion mechanism that has been analyzed in great detail.

### 3.1. Ponderomotive Force Accelerated Transmission of Ceramic Particles in Solid Ion Plasma

The pondermotive force theory in solid state ionic plasmas was developed following a series of theoretical and experimental investigations by Rybakov and Booske et al. [33,34,35,36,37]. In analogy to the plasma counterpart, the pondermotive force is a single particle effect in a spatially varying high-frequency E-field. It is proportional to the gradient of E^2^ while independent of the sign of the charge; thus, electrons and ions move in the same direction to result in a mass flow.

**Figure 7:**
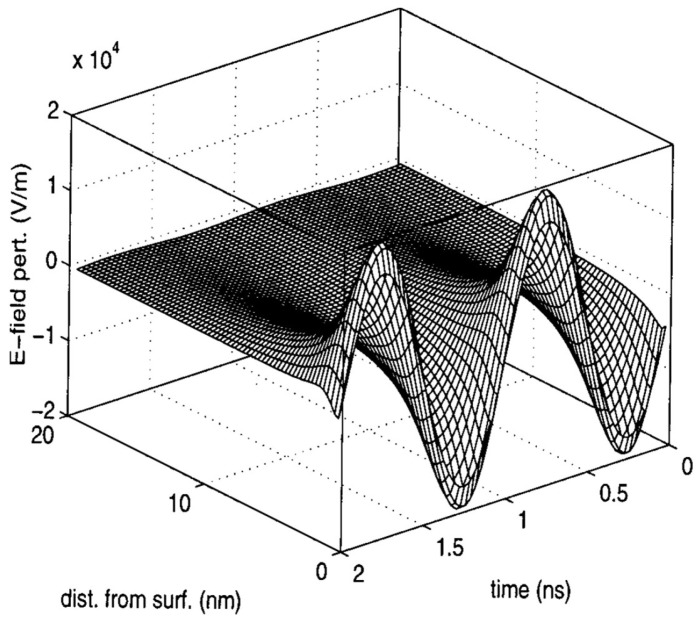
Temporal and spatial dependence of the high-frequency E-field perturbation near the crystal surface [33]. Reproduced with permission from AIP Publishing, 2025.

In a high-frequency microwave, the perturbed charge density will, in general, be localized to the solid surface (Figure 7). The ponderomotive force thus mainly leads to surface mass transport. According to the authors’ theoretical and experimental analysis, in practical conditions, it can have a strength comparable to the conventional driving forces in ceramic and glass processing [33]. Furthermore, a later theory indicates the transport rate can be orders-of-magnitude greater due to the local electric field intensification (see Section 3.3.3). In light of the lack of convective mixing in solids, this is a significant complimentary force to enhance the mass transport in solids.

**Figure 8:**
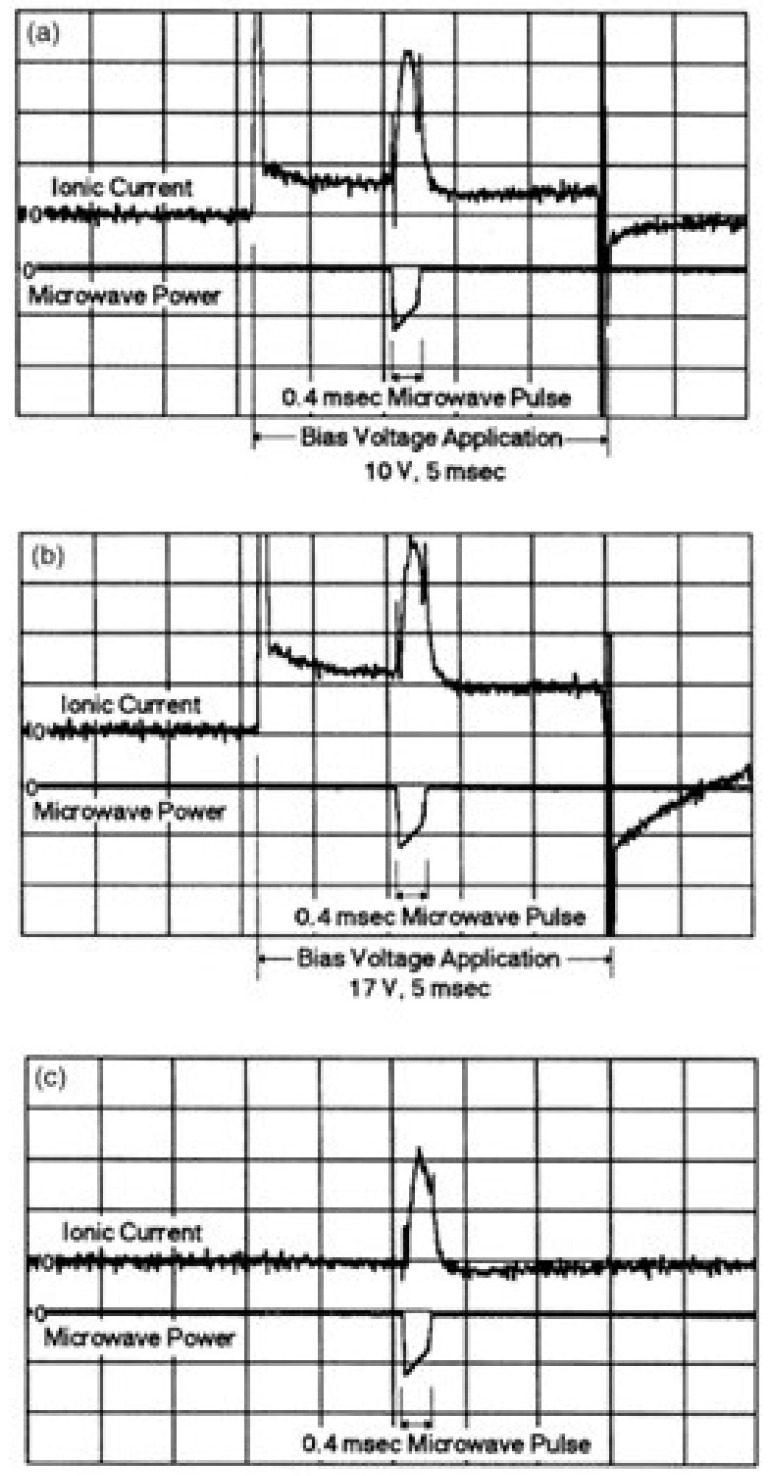
Observed influence of the bias voltage on the ionic current in a 150 °C NaCl crystal: In (**a**), a 10 V, 5 ms bias pulse (1 ms/div) produces an ionic current (0.1 nA/div). In (**b**), a slightly higher ionic current at a 17 V bias voltage. In (**c**), the ionic current vanishes at zero bias voltage. The 2 kW, 0.4 ms microwave-driven current remains at the same level in all three cases. Reproduced with permission from the American Physical Society and SciPris, 2025.

Freeman et al. [34] carried out an experiment that verified the pondermotive force theory. They pulsed a 14 GHz intense microwave for 0.4 ms through a NaC1 crystal sample maintained at 150 °C and also applied an external bias voltage to drive an ionic current. Figure 8a–c show the measured ionic currents at the same microwave power (~2 kW) during its 0.4 ms application, while a bias voltage of 10 V (Figure 8a), 17 V (Figure 8b), and 0 V (Figure 8c) was applied for 5 ms. It can be observed that the bias voltage induced current varies with the bias voltage and vanishes as it is turned off. However, the incremental current during the microwave pulse remains the same. The authors also found that the microwave-driven incremental current was linearly proportional to the microwave power (or *E*^2^). This provides convincing evidence that the incremental current originated from the ponderomotive force.

### 3.2. Magnetic Causes

**Figure 9:**
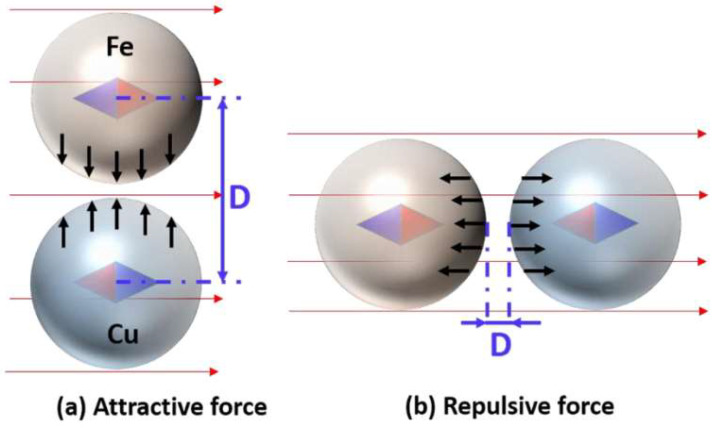
Direction of the magnetization (diamond arrows) of Fe and Cu particles under a uniform H-field and the magnetic force between them. The H-field is (**a**) perpendicular to and (**b**) parallel to the line connecting the particle centers [38].

The magnetic field can also affect microwave sintering [38,39,40]. Xiao et al. [38] modeled Cu–Fe coupled particles in an applied H-field (Figure 9). A magnetic substance forms a small magnetic dipole moment parallel or anti-parallel to the magnetizing field, depending on its magnetic susceptibility. Copper is diamagnetic, while iron is ferromagnetic. So the two particles are magnetized in opposite directions (diamond arrows). A magnetized particle experiences a force only in a spatially varying H-field, so the uniform applied H-field exerts no force on the particle. However, the two magnetized particles each generate a non-uniform self-field, behaving like two magnets exerting a force on each other.

Figure 9 illustrates the magnetic force in two relative positions of the particles. The force is attractive when the applied H-field perpendicular to the line connecting the particle centers Figure 9a and repulsive in a parallel applied H-field Figure 9b. However, the strongest self-field gradient is along the direction of the magnetization. In Figure 9a, the two particles are in each other’s weak self-field gradient, while, in Figure 9b, the two particles are in each other’s strong self-field gradient. This results in a stronger repulsive force.

**Figure 10:**
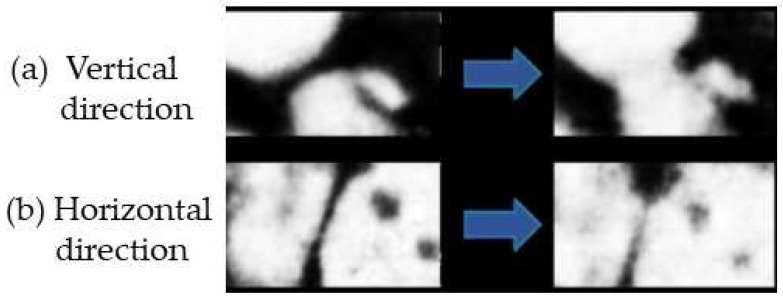
Evolution of the Cu–Fe necks for (**a**) vertical orientation and (**b**) horizontal orientation of the H-field before and after microwave sintering [38].

This model predicts an orientational effect of the H-field on the neck growth between two iron particles. Experimental evidence was provided by the same authors. Figure 10 shows the microstructure evolution of the Cu–Fe neck before and after microwave sintering for vertical orientation (Figure 10a) and horizontal orientation (Figure 10b) of the H-field. The sintering neck is seen to grow large in the former case (Figure 10a), while the growth is restrained in the latter case (Figure 10b).

The wave H-field inside the non-permeable, ceramic particles can induce a circular polarization current which, acted on by the wave H-field, exerts an inward magnetic force. This force, proposed by Xu et al., is referred to as a polarization Ampere’s force [39].

**Figure 11:**
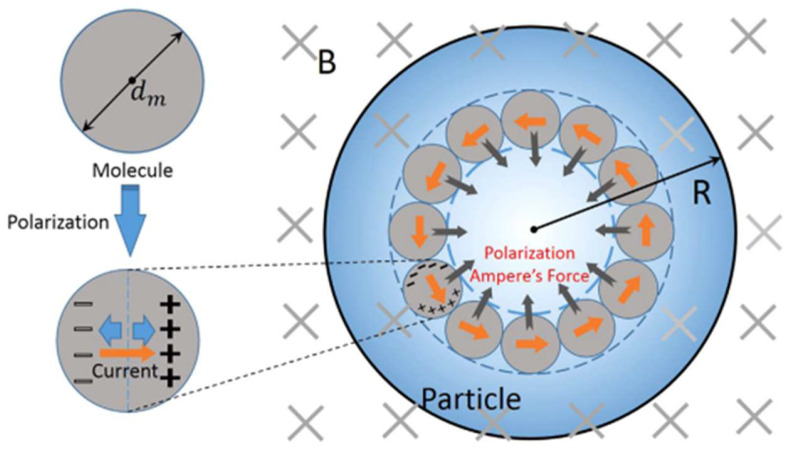
Schematic showing the formation of the polarized Ampere’s force [39]. Reproduced with permission from Elsevier, 2025.

In Xu’s model, as shown in Figure 11, a circular ring of ceramic particles is acted on by a uniform, time-varying AC H-field (pointing into the figure). By Faraday’s law, a circular AC E-field is induced, which then drives a polarization current along the AC E-field. This current and the wave H-field thus produce an inward magnetic force.

**Figure 12:**
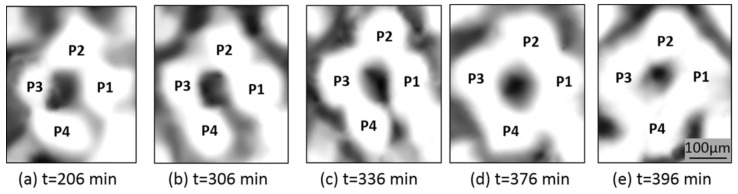
Particle size homogenization in typical regions of the experimental alumina sample as the sintering time increases, [39]. Reproduced with permission from Elsevier, 2025.

Xu’s theory is evidenced by, as well as explains, the experimental images of the microwave sintered alumina sample obtained by the same authors. The inward magnetic force suggests the shrinkage of large particles and expansion of small ones until all have reached approximately equal size. This results in a particle homogenization phenomenon, as shown in the microstructure evolution in Figure 12. In Figure 12a, four alumina particles (labeled with P1–P4) have various sizes before sintering, with P3 as the smallest. As the microwave was turned on, P3 gradually grew, while the large particles (P1, P2, and P4) became smaller. Finally, the four particles approach approximately the same size (Figure 12b–e).

**Figure 13:**
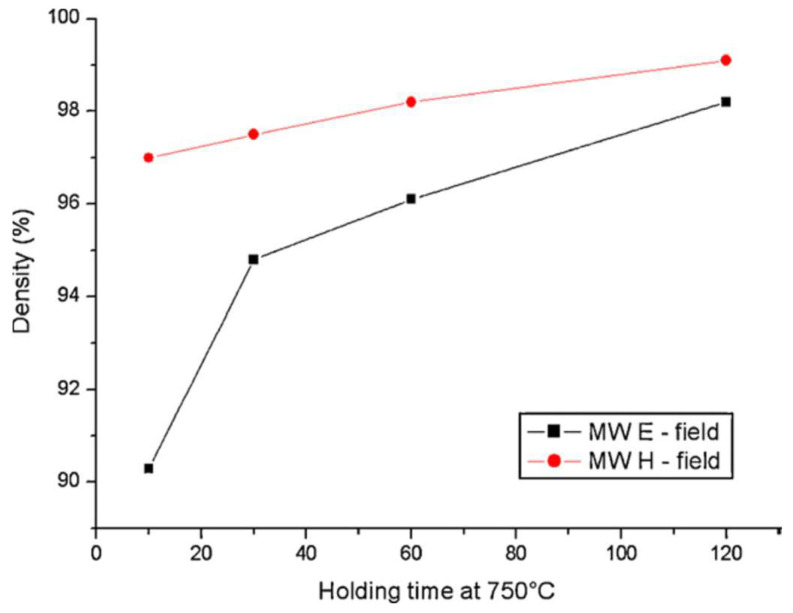
Density variation ZnO samples versus the holding time at 750 °C under the microwave E*-* and H-field [40]. Reproduced with permission from Elsevier, 2025.

In a study to compare the influence of wave E- and H-fields on semiconductor sintering, Badev et al. [40] studied the ZnO densification in the E- and H-field regions of a single-mode cavity in identical heating cycles. The results show different behaviors, with better densification in the H-field (Figure 13). The authors attributed the difference to an electromagnetic pressure induced by the combined effect of current loops and the wave H-field. The magnetic pressure in turn leads to better contact between particles and therefore enhances the diffusion.

### 3.3. Enhanced Reaction in Polarization Charge-Induced Electric Field

#### 3.3.1. Formation of Polarization Charges

The presence of an E-field will slightly displace the electrons tightly bound to the molecules. Inside the dielectric, a displaced electron will be replaced by another displaced electron. So, the electron displacements will not (or barely) change the inside charge density. However, the replacement is not possible on the surfaces. So the electrons become excessive on one surface to form negative “polarization charges” and partially depleted on the opposite surface to form positive polarization charges.

**Figure 14:**
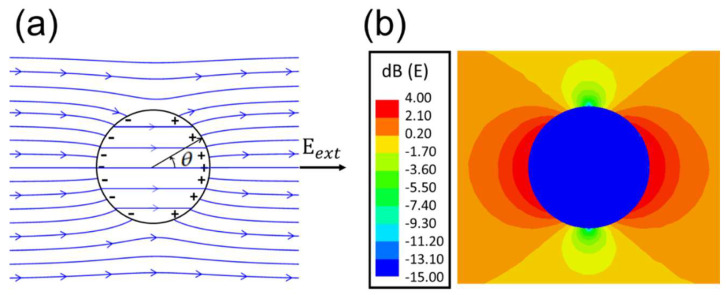
(**a**) E-field lines of a dielectric sphere of *ε/ε*_0_ =4 in the presence of a static and uniform external *E_ext_* as calculated from Equation (1). Surface polarization charges are seen to be induced. (**b**) For a much larger *ε/ε*_0_ = 80, the simulated *E(*x*)* displays a much greater variation with relative amplitude given by the color code [42]. Reproduced with permission from AIT Publishing, 2025.

Consider the simple case of a uniform sphere in a static, uniform *E_ext_*. Induced surface polarization charges (*σ_pol_*) on the spherical surface are given by Ref. [41] (Section 4.4).

(1) $$\sigma_{pol}=3\varepsilon_{0}(\frac{\varepsilon -\varepsilon_{0}}{\varepsilon +2\varepsilon_{0}})E_{ext}\cos\theta \;$$

where *ε* and *ε*_0_ are the sphere and vacuum permittivity, and *θ* is the observation angle in Figure 14a.

The *±σ_pol_* partially cancels the *E_ext_* inside the sphere (Figure 14a) while still maintaining a uniform interior field (*E_in_*). The cancelation is larger for a larger dielectric constant. For example, *E_in_*/*E_ext_* = 0.5 for ε/ε_0_ = 4 and *E_in_*/*E_ext_* = 0.037 for *ε/ε*_0_ = 80. Figure 14a plots the directions and discontinuities (at *σ_pol_*) of E-field lines for *ε/ε*_0_ = 4, while Figure 14b shows the large field variations for *ε/ε*_0_ = 80 in color code.

Equation (1) describes a static case valid for any sphere radius. In the presence of an electromagnetic wave of free-space wavelength *λ_fre_*, the wavelength will be *λ_d_* [=*λ_free_*/Re(*ε/ε*_0_)] in the dielectric sphere. If a sphere of radius *R* is in the near zone if *λ_d_* >> *R*, the induced, oscillating E-field is “quasi-static”; namely, it has a spatial profile closely approaching that of the static case in Figure 14 (Ref. [41], Section 10.1).

#### 3.3.2. Theory of Electric Field Intensification by Polarization Charges

**Figure 15:**
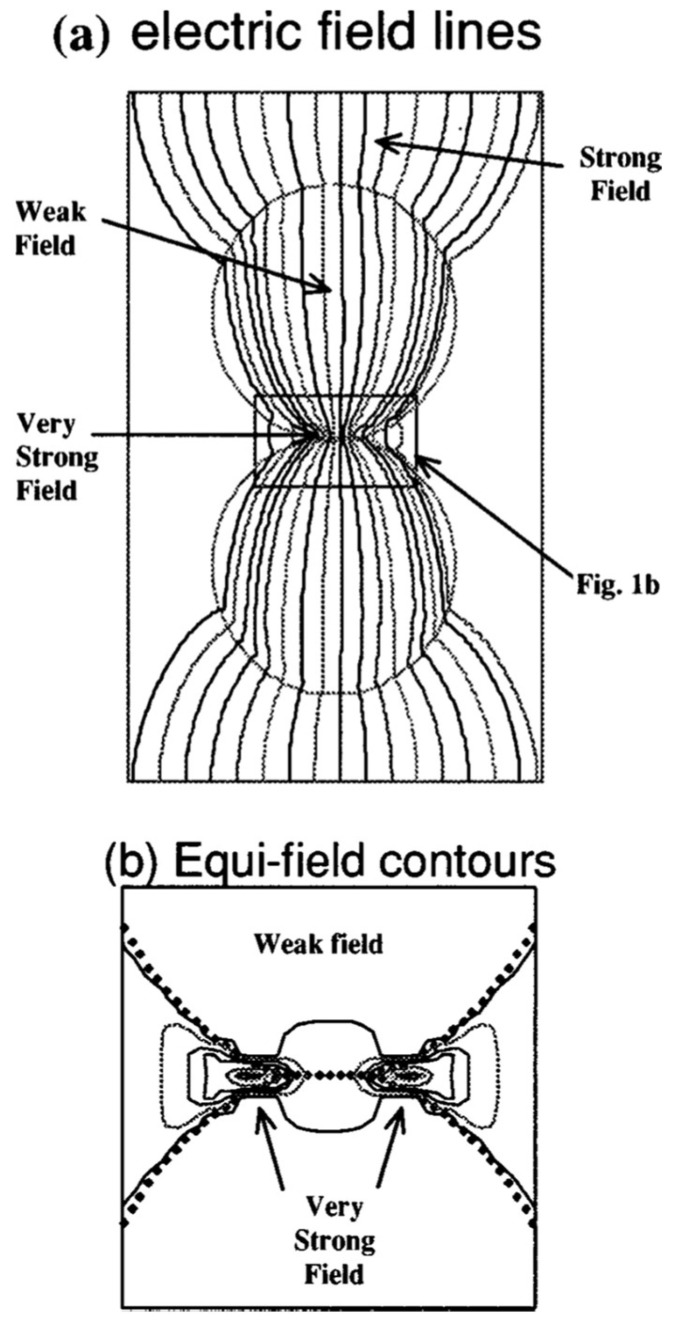
(**a**) The E-field concentrates in the gap of two dielectric spheres in the presence of *E_ext_*. (**b**) Close-up of the isoelectric field pattern in the gap [43]. Reproduced with permission from AIT Publishing, 2025.

Calculations by Birnboim et al. [43] have shown that, when two dielectric spheres that nearly touch are aligned along *E_ext_*, an E-field >> *E_ext_* is induced in the gap region (Figure 15). Quantitatively, depending on the dielectric constant, the authors found that the peak E-field is ~30 times higher than *E_ext_* for Z_n_O spheres and ~14 times higher than *E_ext_* for Al_2_O_3_ spheres.

**Figure 16:**
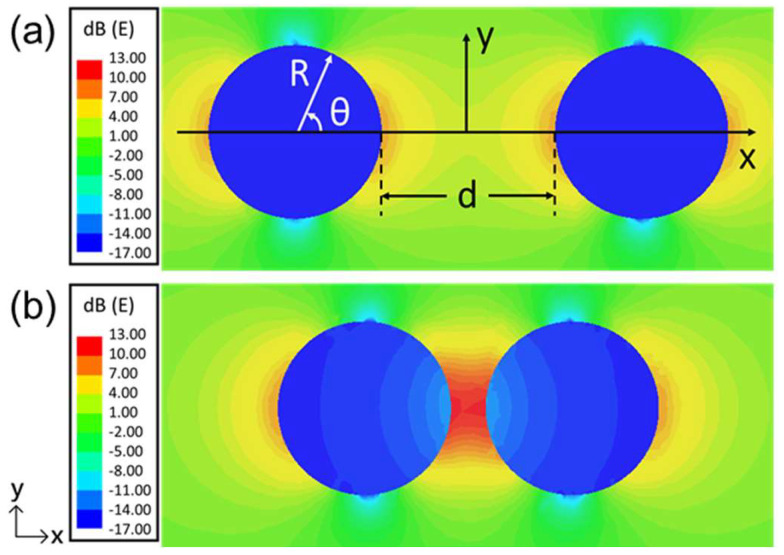
Illustration of *σ_pol_* buildup and *E_gap_* (at *z* = 0) intensification between two identical dielectric spheres of radius *R*, separation *d* and *ε* = 20*ε*_0_ in a uniform, static, and linearly polarized, 2.45 GHz *E_ext_***e**_x_. (**a**) *d* = 2*R* and (**b**) *d* = 0.4*R*. The relative E-field strength (normalized to *E_ext_*) is given by the color code [44].

Lin et al. [42] and Liu et al. [44] further studied the gap E-field intensification phenomenon. They show that, when two dielectric spheres (of radius *R*) are put closer to each other, the positive and negative polarized charges on opposite sides of their gap surfaces *(±σ_pol_*) will induce more *±σ_pol_*, leading to an increased gap E-field (*E_gap_*) (Figure 16).

**Figure 17:**
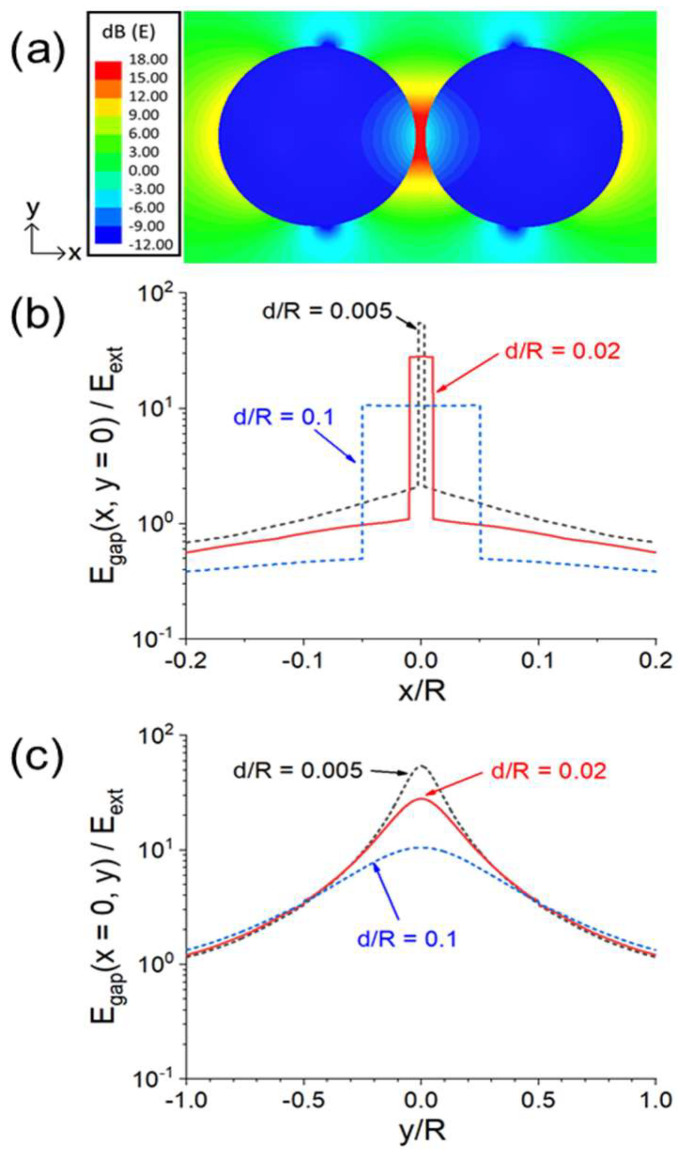
Quantitative illustration of *E_gap_* intensification of the two spheres in Figure 16: (**a**) E(*z* = 0)-field pattern plotted on the *x-y* plane for *d* = 0.1*R*. (**b**) *E_gap_* (*x, y* = 0)/*E_ext_* versus *x* for *d* = 0.005*R*, 0.02*R*, and 0.1*R*. (**c**) *E_gap_* (*x*= 0, *y*)/*E_ext_* versus *y* for *d* = 0.005*R*, 0.02*R*, and 0.1*R* [44].

**Figure 18:**
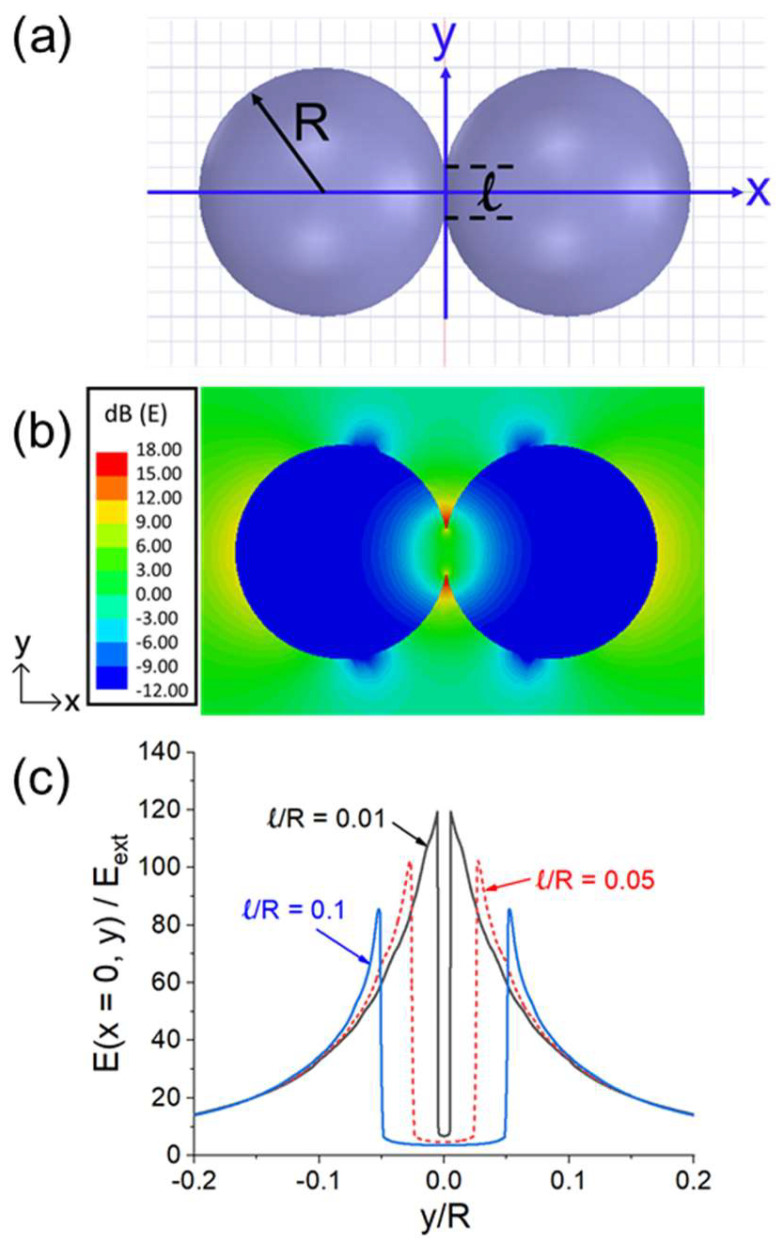
Quantitative illustration of *E_gap_* intensification when the two spheres are connected over a neck length of *ℓ* [as shown in (**a**)]. (**b**) E(*z* = 0)-field pattern on the *x-y* plane for *ℓ* = 0.4*R*. (**c**) E (*x* = 0, *y*) versus *y* for *ℓ* = 0.01*R*, 0.05*R*, and 0.1*R* [44].

The enhanced E-field under a 2.45 GHz microwave as a function of the sphere separation *d* is shown in Figure 17b. *E_gap_* becomes larger as *d* becomes smaller. As shown in Figure 17c, when two dielectric particles are in contact, there is still an increase in *E_gap_* when viewed from the *y* direction. A similar E-field intensification effect is shown in Figure 18 for two spheres connected over a neck length of *ℓ*.

#### 3.3.3. Effect of Enhanced Electric Field on Microwave Sintering

From their result shown in Figure 15, Birnboim et al. [43] found that the intensified *E_gap_* between two contacting dielectric spheres is 14–30 times higher than the wave E-field. According to the ponderomotive force theory, the mass flow rate scales with *E*^2^. Thus, the flow rates can be greatly enhanced due to the local *E_gap_* intensification.

**Figure 19:**
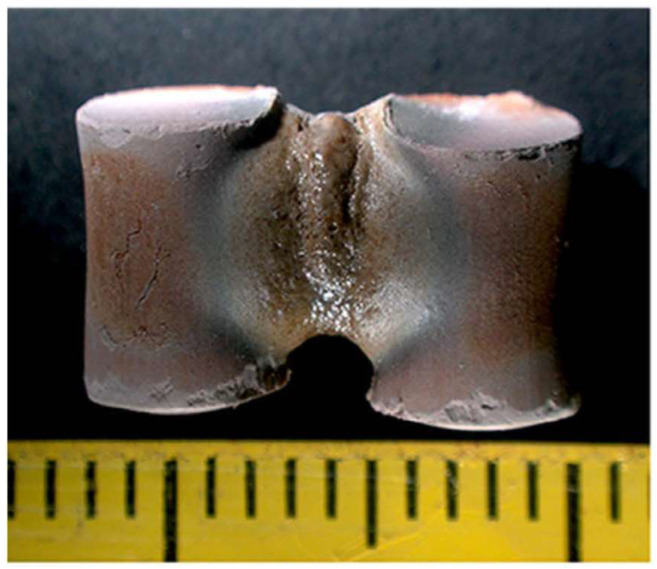
Two CFA cylinder samples sintered by 250 W microwave for 100 s [45].

The prediction by Birnboim et al. appears to be corroborated by Qiao and Xie, who showed, in simulation and experiment, that the intensified *E_gap_* led to the fusing of solid materials [coal fly ash (CFA) and copper] and enhanced the mass transport [45]. In their experiment, two CFA cylinders (1.4 × 10 mm) were sintered by an intensified *E_gap_* of 7.84 × 10^3^V/cm (calculated) perpendicular to the contact boundary when exposed to a 250 W microwave for 100 s. A large amount of the fused phase was seen to form in their gap (Figure 19).

## 4. A Recent Theory on Electric Force Attraction Due to Polarization Charge Enhancement

As discussed in Section 3.3, an intensified *E_gap_* is expected to greatly promote mass transport in microwave sintering. On the other hand, there is also an attractive force between the spheres due to the action of *E_gap_* on *±σ_pol_*. The relevant question here is whether this force is strong enough to also play a positive role in microwave sintering. This seems to be highly likely based on the calculations below.

### 4.1. Force Density on the Spherical Surface

**Figure 20:**
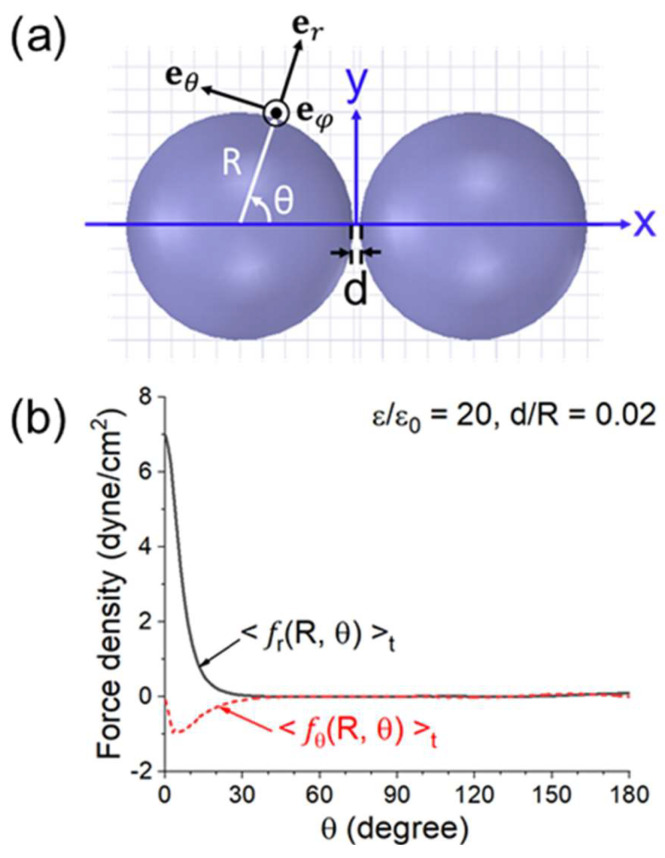
(**a**) Configuration and coordinate system for the calculation. (**b**) *r*− and *θ*− components of the force density on the left sphere versus *θ* for *ε* = 20*ε*_0_, *d* = 0.02*R* and *E_ext_* (peak) = 200 V/cm [44].

The *E_gap_* and ±*σ_pol_* will produce an attractive force between the two spheres. By symmetry, the forces on both spheres are equal in amplitude, so we only need to consider the left sphere. Assume its center is located at *r* = 0 in spherical coordinates (Figure 20a). By symmetry, the E-field is independent of the azimuthal angle *φ*, and *σ_pol_* depends only on *θ*. With a negligible displacement current in the quasi-static regime, we have *E_φ_* = 0. Note that a charge’s self-field does not exert a force on itself. It can be shown [46] that the effective field (**E***_eff_*) acting on *σ_pol_* is [46] (all equations below are reproduced from the authors’ earlier publication [44]):

(2) $$\begin{array}{ll} E_{eff}(R,\theta ) & =\frac{1}{2}[E(R^{-},\theta )+E(R^{+},\theta )]\\ & =\frac{1}{2}[E_{r}(R^{-},\theta )+E_{r}(R^{+},\theta )]e_{r}+E_{\theta }(R,\theta )e_{\theta }\; \end{array}$$

where *R^±^ = R ± δ* with *δ*→0. *E_r_* is discontinuous across the surface due to *σ_pol_* at *r* = *R*; hence, the second equality in Equation (2).

Since there is no free charge, we have a continuous electric displacement, i.e., *ε*_0_*E_r_*(at *r = R^+^*) = ε*E_r_*(at *r* = *R^−^*):

(3) $$E_{r}(R^{-},\theta )=E_{r}(R^{+},\theta )\frac{\varepsilon_{0}}{\varepsilon }\;$$

By Equations (2) and (3), we obtain

(4) $$E_{eff}(R,\theta )=\frac{E_{r}(R^{+},\theta )}{2}(1+\frac{\varepsilon_{0}}{\varepsilon })e_{r}+E_{\theta }(R,\theta )e_{\theta }\;$$

Applying Gauss law and Equation (3) gives *σ_pol_*

(5) $$\sigma_{pol}(R,\theta )=\varepsilon_{0}(1-\frac{\varepsilon_{0}}{\varepsilon })E_{r}(R^{+},\theta )\;$$

In the quasi-static regime, *σ_pol_*, *E_r_*, and *E_θ_* are all oscillating in phase. So, their time-average is simply one half of their peak values. Thus, the force per unit area (force density) is

(6) $$\begin{array}{l} {\langle f(R,\theta )\rangle }_{t}={\langle \sigma_{pol}(R,\theta )E_{eff}\rangle }_{t}\\ =\frac{1}{4}\varepsilon_{0}(1-\frac{\varepsilon_{0}^{2}}{\varepsilon^{2}})E_{r}^{2,\mathrm{peak}}(R^{+},\theta )e_{r}+\frac{1}{2}\varepsilon_{0}(1-\frac{\varepsilon_{0}}{\varepsilon })E_{r}^{peak}(R^{+},\theta )E_{\theta }^{peak}(R,\theta )e_{\theta } \end{array}$$

where the peak value (in time) of a quantity is denoted by the superscript “peak”. In our linear model, *E_r_* and *E_θ_* scale linearly with *E_ext_* and depend on *d/R* (or *ℓ/R*).

**Figure 21:**
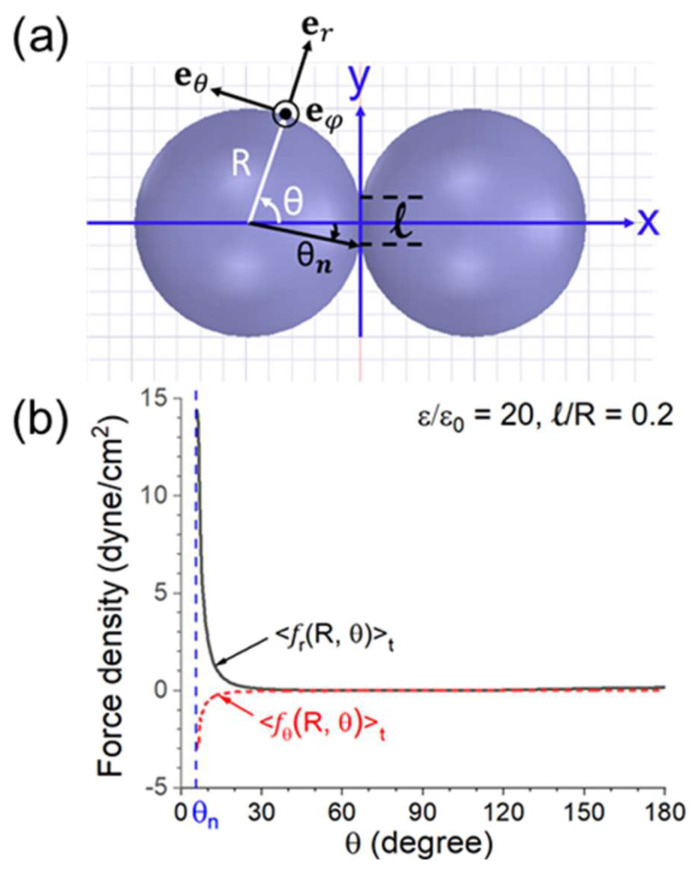
(**a**) Configuration and coordinate system for the calculation. (**b**) *r*− and *θ*− components of the force density on the left sphere versus *θ* for *ε* = 20*ε*_0_, *ℓ* = 0.2*R*, and *E_ext_* (peak) = 200 V/cm [44].

The *r*- and *θ*-components of Equation (6) are, respectively, <*f_r_* (*R*, *θ*)>*_t_* and <*f_θ_* (*R*, *θ*)>*_t_*. The two components (in dyne/cm^2^) versus *θ* are illustrated in Figure 20 for *ε* = 20*ε*_0_, *d* = 0.02*R*, and *E_ext_*(peak) =200 V/cm (as in a microwave oven [47]). <*f_r_* (*R*, *θ*)>*_t_* peaks sharply at *θ* ≈ 0. The same quantities for two connected spheres with *ε*= 20*ε_0_* and *ℓ* = 0.2*R* are shown in Figure 21. In Figure 21b, the force only appears at *θ* > *θ_n_*, where *θ_n_* is the neck’s angular half width. As in Figure 20, <*f_r_* (*R*, *θ*)>*_t_* has a sharp and narrow peak of at *θ* ≈ *θ_n_*.

**Figure 22:**
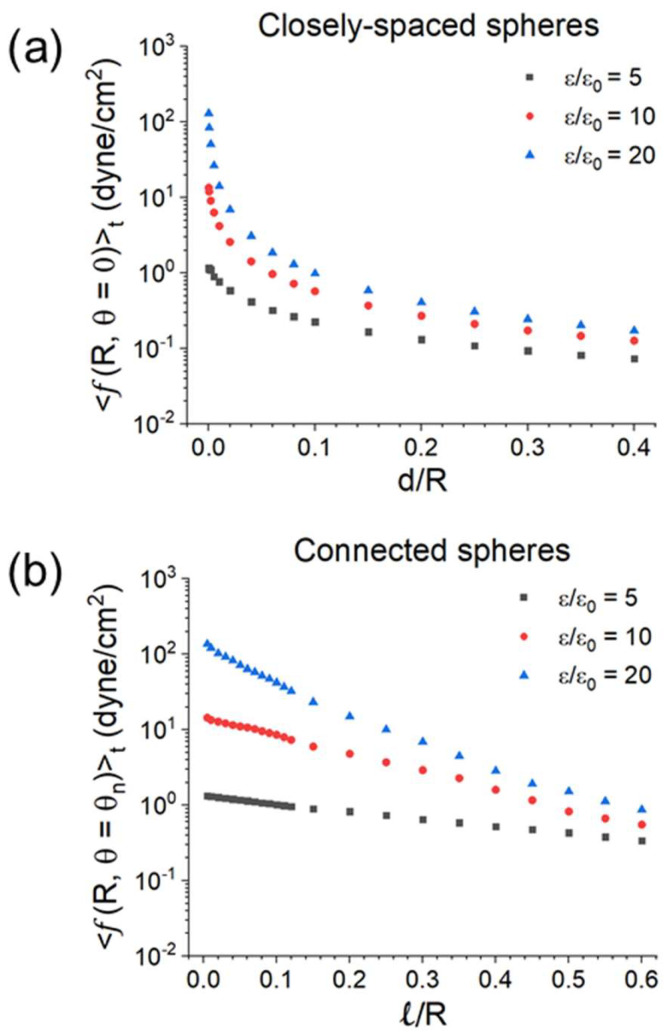
(**a**) The maximum force density <*f*(*R*, *θ* = 0)>*_t_* (with respect to *θ*) versus *d/R* for two closely spaced spheres; and (**b**) the maximum force density <*f*(*R*, *θ* = *θ_n_*)>*_t_* versus *ℓ/R* for two connected spheres. *ε/ε*_0_ = 5, 10, 20, and *E_ext_* (peak) = 200 V/cm [44].

Assume *ε/ε*_0_ = 5, 10, 20, and *E_ext_* (peak) = 200 V/cm. The maximum of *f* with respect to *θ* is at *θ* = 0 or *θ_n_*. Figure 22 displays <*f*(*R*, *θ* = 0)>*_t_* versus *d/R* for two closely spaced spheres and <*f*(*R*, *θ* = *θ_n_*)>*_t_*versus *ℓ/R* for two connected spheres. In both cases, the maximum force density for *ε/ε_0_* = 20 peaks at ~130 dyne/cm^2^ with a corresponding E ≈ 2.5 × 10^4^ V/cm (below the air breakdown field of 3 × 10^4^ V/cm at 1 atm). The region of larger *f* (*θ* = 0 or *θ_n_*) is where powder compaction and neck growth take place.

### 4.2. Total Attractive Force

By symmetry, the total attractive force is the x-component of <**f**(*R*, *θ*)>*t*

(7) $$\begin{array}{l} {\langle f_{x}(R,\theta )\rangle }_{t}={\langle f(R,\theta )\rangle }_{t}\cdot e_{x}\\ =\frac{1}{4}\varepsilon_{0}(1-\frac{\varepsilon_{0}^{2}}{\varepsilon^{2}})E_{r}^{2,\mathrm{peak}}(R^{+},\theta )\cos\theta \\ -\frac{1}{2}\varepsilon_{0}(1-\frac{\varepsilon_{0}}{\varepsilon })E_{r}^{\mathrm{peak}}(R^{+},\theta )E_{\theta }^{\mathrm{peak}}(R,\theta )\sin\theta \; \end{array}$$

We now limit our consideration to two closely spaced spheres. Their total attractive force is given by an integration of Equation (7) over the spherical surface

(8) $${\langle F_{x}(R,\theta )\rangle }_{t}=\oint_{s}{\langle f_{x}(R,\theta )\rangle }_{t}da=2\pi R^{2}\int_{0}^{\pi }{\langle f_{x}(R,\theta )\rangle }_{t}\sin\theta d\theta$$

which is to be evaluated numerically. Figure 20 and Figure 21 show that force density is primarily in the radial direction, which depends on the square of *E_r_*_._ Equation (6). Hence, the total force in Equation (8) is independent of the phase of *E_r_*, i.e., always attractive.

Equation (8), derived for *E_ext_***e**_x_, gives a total force in the x-direction. A randomly polarized *E_ext_* will produce an isotropic total force, with half of the value in Equation (8).

The surface area of a sphere scales with *R*^2^, while its mass scales with *R*^3^. Thus, a smaller sphere is accelerated more in the same *E_ext_*. We define the force per unit volume as

(9) $$\frac{\mathrm{force}\;}{\mathrm{unit}\;\mathrm{volume}}=\frac{\mathrm{total}\;\mathrm{force}\;\mathrm{on}\;\mathrm{sphere}}{\mathrm{volume}\;\mathrm{of}\;\mathrm{sphere}}=(\frac{3}{4\pi R^{3}}){\langle F_{x}(R,\theta )\rangle }_{t}$$

**Figure 23:**
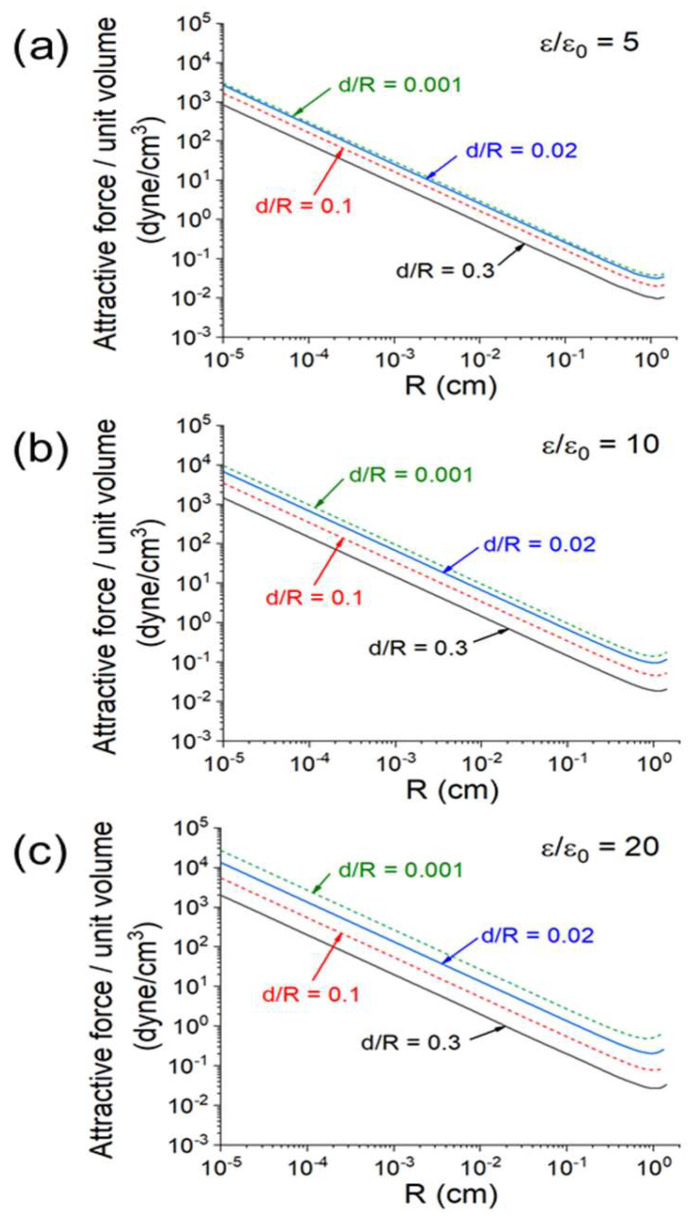
Attractive force per unit volume versus the sphere radius *R* under *E_ext_*(peak) = 200 V/cm for four values of *d/R*. (**a**) *ε/ε*_0_ = 5, (**b**) *ε/ε*_0_= 10, and (**c**) *ε/ε*_0_ = 20 [44].

The size (*R*) dependence of the attractive force per unit volume in Equation (9) is plotted in Figure 23 for *E_ext_* (peak) = 200 V/cm in a broad space of *d/R* and *ε/ε*_0_. As an example, an *R* = 1 μm sphere 2.5 in specific weight will be accelerated up to a few g (=980 cm/s^2^) in *E_ext_*(peak) = 200 V/cm.

### 4.3. Significance of the Attractive Force to Microwave Sintering

As discussed earlier, the electric force always produces an attractive force in the oscillating *E_ext_*. Compared with the *contact force* under an externally applied pressure, the E-field produces a *non-contact force*. The starting powder is usually formed of nearly spherical particles. For two closely spaced spheres 1 μm in radius and 2.5 in specific weight under *E_ext_* (peak) = 200 V/cm, the attractive force can accelerate the particles up to a few g (=980 cm/s^2^) (Figure 23), which is greater than or comparable to gravitational or centrifugal compaction. As a neck is formed between the two spheres, the force still acts on the gap near the neck at a strength up to 100 dyne/cm^2^ (Figure 22b). It thus continues to promote the densification process, particularly in the liquid phase.

### 4.4. A Relevant Experiment

**Figure 24:**
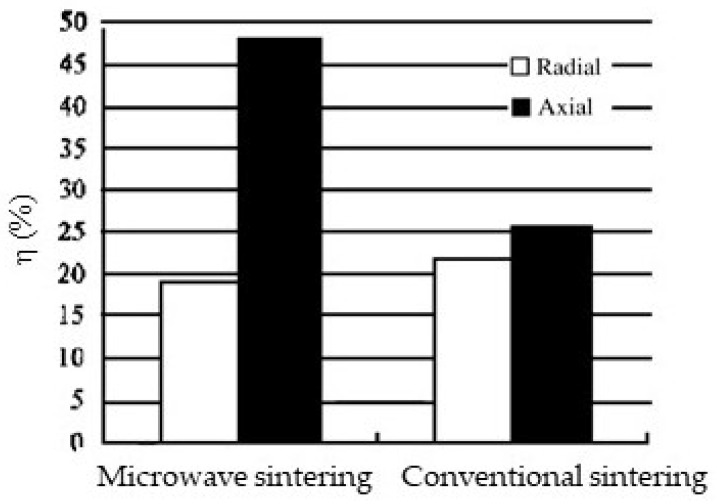
Comparison of the shrinkage rate (*η*) of ceramic samples between furnace and microwave sintering [48]. Reproduced with permission from Elsevier, 2025.

**Figure 25:**
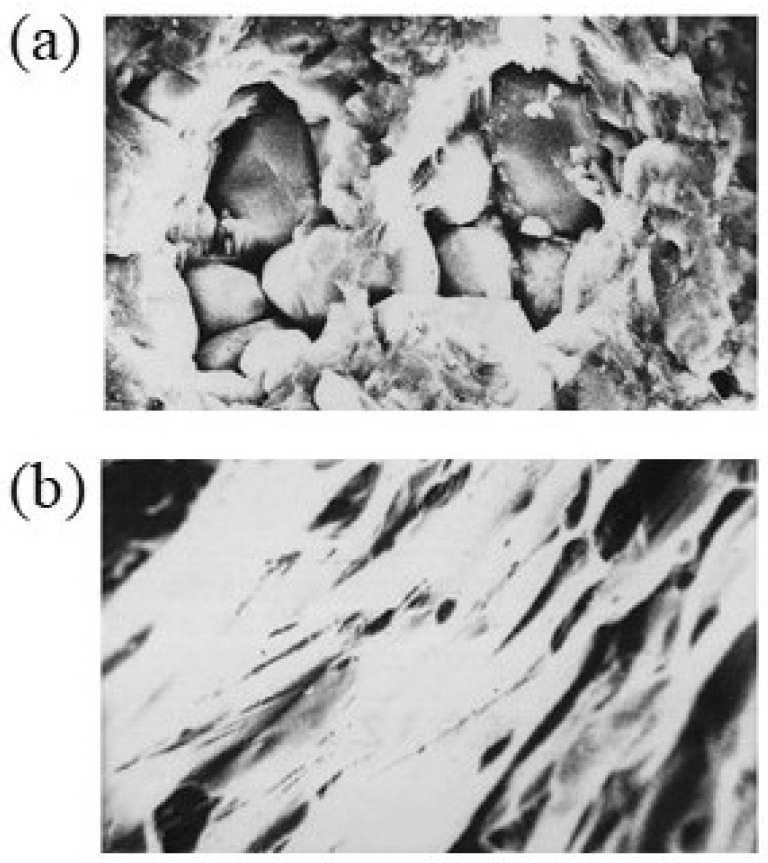
The sample microstructure in (**a**) furnace sintering and (**b**) microwave sintering [48]. Reproduced with permission from Elsevier, 2025.

Chang and Jian presented a study focusing on the orientational effect [48]. They sintered a doped BaTiO_3_ PTCR sample cylindrical in shape with a 2.45 GHz microwave linearly polarized along the axis of the cylinder. The treatment was at 1250 °C for 15 min. For comparison, they also sintered an identical sample at 1350 °C for 2 h in a furnace. In microwave sintering, the sample shrinkage rates and grain growth showed a strong orientational dependence. The maximum axial shrinkage rate is 48% in the microwave as compared to 26% in a furnace (Figure 24). In comparison, the radial shrinkage rate showed little difference between the two sintering methods. In addition, the microwave-sintered grains also exhibit a strip-like microstructure along the E-field (Figure 25).

The reason for Chang and Jian’s observations is still unclear. However, a physical effect along the E-field has clearly taken place. The directional coincidence of the maximum shrinkage rate and maximum *σ_pol_* buildup suggests the attractive force to be a plausible cause, although more verifying experiments are needed.

## 5. Conclusions

As surveyed in Section 2, microwave sintering has been widely shown in experiments to require a substantially lower processing temperature to achieve the same degree of densification as in furnace sintering. The reason for this benefit is subject to different physical interpretations depending on the type of sintered materials. It is referred to as a non-thermal effect; namely, it is due to either the wave electric or magnetic field. Theoretical studies on these observations have shed much light on the non-thermal effect. However, the proposed causes largely remain an open issue. A definitive investigation of the non-thermal effect is thus much needed in order to bring this promising but not yet fully realized technology to fruition.

To promote and facilitate further research, Section 3 sorts the main points out of a wide spread of the literature on the potential causes proposed in early and recent theories for the non-thermal effect and illustrate each one with representative figure(s) from the original publication, with an emphasis on the latest development.

In Section 4, we discuss in greater detail the polarization charge intensification effect, which can greatly reinforce the early proposed mass flow driven by the ponderomotive force, while also leading to an attractive force between neighboring particles. The attractive force concentrates in the region where the primary sintering process takes place. As quantitatively illustrated in Figure 23, the force has a strength significant enough to assist particle compaction and neck growth. It also explains the directional effect found in an early experiment, which showed a much greater sample shrinkage rate along the wave E-field.

The main limitation of future works lies in the complexity of material behavior and the difficulty of experimental diagnostics in penetrating through the material’s interior to verify a proposed theory. Nevertheless, the formalism and data base presented here could hopefully be useful for further studies to understand the long-standing question of microwave-enhanced densification.

## Figures and Tables

**Table 1:** Metallic powders of the designed recipe for FeCuCo matrix [22]. Reproduced with permission from Elsevier, 2025.

Elements	Fe	Cu	Co	Sn	Ni
Content (w.t.%)	40	30	13	7	10
Average particle size (μm)	31.7 μm	23.1 μm	20.4 μm	21.5 μm	20.8 μm
Purity (w.t.%)	>99.5	>99.7	>99.9	>99.9	>99.8

## Data Availability

No new data were created or analyzed in this study. Data sharing is not applicable to this article.
